# The Orally Available, Synthetic Ether Lipid Edelfosine Inhibits T Cell Proliferation and Induces a Type I Interferon Response

**DOI:** 10.1371/journal.pone.0091970

**Published:** 2014-03-25

**Authors:** Pierre Abramowski, Benjamin Otto, Roland Martin

**Affiliations:** 1 Institute for Neuroimmunology and Clinical Multiple Sclerosis Research (inims), ZMNH, University Medical Center Hamburg-Eppendorf, Hamburg, Germany; 2 Research Department Cell and Gene Therapy, Clinic for Stem Cell Transplantation, University Medical Center Hamburg-Eppendorf, Hamburg, Germany; 3 I. Department of Internal Medicine, Center for Internal Medicine, University Medical Center Hamburg-Eppendorf, Hamburg, Germany; 4 Department of Clinical Chemistry, Center for Diagnostic, University Medical Center Hamburg-Eppendorf, Hamburg, Germany; 5 Neuroimmunology and MS Research, Department of Neurology, University Hospital Zurich, Zurich, Switzerland; University Hospital of Heidelberg, Germany

## Abstract

The drug edelfosine is a synthetic analog of 2-lysophosphatidylcholine. Edelfosine is incorporated by highly proliferating cells, e.g. activated immune cells. It acts on cellular membranes by selectively aggregating the cell death receptor Fas in membrane rafts and interference with phosphatidylcholine (PC) synthesis with subsequent induction of apoptosis. Edelfosine has been proposed for the treatment of autoimmune diseases like multiple sclerosis (MS). Earlier studies on the animal model of MS, experimental autoimmune encephalomyelitis (EAE), have generated first evidence for the efficacy of edelfosine treatment. However, it is unknown if the previously described mechanisms for edelfosine action, which are derived from *in vitro* studies, are solely responsible for the amelioration of EAE or if edelfosine may exert additional effects, which may be beneficial in the context of autoimmunity. Since it was the purpose of our studies to assess the potential usefulness of edelfosine for the treatment of MS, we examined its mechanism/s of action on immune functions in human T cells. Low doses of edelfosine led to a decrease in homeostatic proliferation, and further studies of the mechanism/s of action by genome-wide transcriptional profiling showed that edelfosine reduces the expression of MHC class II molecules, of molecules involved in MHC class II-associated processing and presentation, and finally upregulated a series of type I interferon-associated genes. The inhibition of homeostatic proliferation, as well as the effects on MHC class II expression and –presentation, and the induction of type I interferon-associated genes are novel and interesting in the context of developing edelfosine for clinical use in MS and possibly also other T cell-mediated autoimmune diseases.

## Introduction

The 2-lysophosphatidylcholine analog edelfosine (1-O-octadecyl-2-O-methyl-rac-glycero-3-phosphocholine, ET-18-OCH_3_) was synthesized in 1969 [1]. In 1979 Andreesen *et al.* already reported that 5 µg/ml edelfosine selectively induced cell death in mitogen-activated human peripheral blood lymphocytes (PBLs) *in vitro*, whereas the viability of resting PBLs was not impaired [2]. Later, edelfosine was shown to selectively induce apoptosis in leukemic cells [3]. In 1999, Cabaner *et al.* examined the mechanisms of immunomodulation by edelfosine [4], and their findings implied that apoptosis induction is not only the main principle leading to the drug's antitumor activity, but might also account for its immunomodulatory effects. In contrast to other cytotoxic drugs, alkyl lysophospholipids (ALPs) do not target the DNA. Edelfosine induces apoptosis by recruitment of Fas/CD95 and subsequent death-inducing signaling complex (DISC) formation in a lipid raft-mediated process thereby exerting its cytotoxic activity in the absence of FasL [5], [6]. Additionally, edelfosine may accumulate in lipid rafts within the plasma membrane followed by endocytosis and translocation to the intracellular location of the CTP:phosphocholine cytidylyltransferase (CCT), the endoplasmic reticulum [7], [8]. Here, edelfosine inhibits the *de novo* biosynthesis of PC and lipid second messenger-based signal transduction pathways leading to mitotic arrest and apoptosis [9]. Thus, ALPs like edelfosine may affect several cellular processes, some probably specifically on certain cell types but with the main outcome of apoptosis induction. The degree of cellular edelfosine uptake and thus apoptosis correlates with the proliferative activity and the associated metabolic lipid turnover in the cell [10]. Therefore, not only tumor cells but also normal cells are sensitive to ALPs provided they are proliferating [11]. Based on these immunomodulatory properties and its excellent safety profile, its oral availability and the capability to cross the blood brain barrier led to further investigation of edelfosine as a potential treatment in autoimmune diseases, for instance MS.

MS is considered a prototypic CD4^+^ T helper cell-mediated demyelinating autoimmune disease of the central nervous system (CNS) [12]. Hallmarks of MS pathology are inflammatory lesions within the CNS, de- and partial remyelination of axons, axonal and neuronal loss and glial scarring [12]. Etiologically, MS is considered a typical complex genetic disorder with multiple variants of genes contributing to MS risk [13], however with small effects at the level of the individual gene. The only exception is the major histocomplatibility complex (MHC in general; HLA in humans) HLA-DR15 haplotype, which has first been described as MS risk factor in 1973 [14] and since then has remained the most important individual risk factor, to which between 10 to 60% of the genetic risk in MS has been attributed [15]. Variations in the two cytokine receptor subunits IL-7RA and IL-2RA and in numerous other genes have been described as additional risk alleles for MS, and interestingly, many of these are involved in T cell activation, -proliferation and -function [16], [17]. While it is currently not clear for most of the known risk genes how they contribute to disease at the functional level, we recently demonstrated that autologous/homeostatic T cell proliferation is elevated in MS, and that this effect is related to the HLA-DR15 haplotype [18]. Established environmental risk factors are Epstein-Barr virus infection [19], low vitamin D levels [20] and smoking [21]. Besides the above genetic and environmental MS risk factors which strongly support the autoimmune nature of the disease, investigators have extensively examined animal models that share similarities with MS, particularly the EAE model [22]. Animal- and human studies point to a central role for autoreactive CD4^+^ T cells in MS pathogenesis.

Consistent with the above data, currently approved MS treatments target the autoimmune inflammation and act as immunosuppressants/immunomodulators. High dose glucocorticoids are used during MS relapses as acute intervention, while the peptidic mixture glatiramer acetate and particularly several formulations of interferon (IFN)-β have been used now for almost two decades as first-line therapies in relapsing-remitting MS. In very active patients or upon failure to respond to first-line drugs, immunosuppressants with unspecific effects, mitoxantrone, or the anti-VLA-4 antibody natalizumab, which specifically inhibits the migration of activated T cells into the CNS, are used to escalate treatment intensity. All the above drugs need to be injected or infused, but recently several oral drugs have been approved. Fingolimod, a sphingosine 1 phosphate receptor agonist, which traps certain T cell populations in secondary lymphoid organs, was the first oral treatment for MS three years ago, and now two additional compounds, teriflunomide and fumaric acid, have been approved. The first-line treatments glatiramer acetate and IFN-β have an overall very benign safety profile, but need to be injected. The more effective natalizumab is also usually well tolerated, but the development of progressive multifocal leukoencephalopathy (PML), an opportunistic infection of the brain with the polyomavirus JC, constitutes a very serious side effect. Fingolimod, although generally safe, has led to macular edema in the eye, cardiovascular side effects, and complications with reactivation of latent herpes virus infections including a few deaths. The safety profile of the recently approved drugs teriflunomide and fumaric acid remains to be determined. When considering the fact that relatively large percentages of patients do not respond or respond only incompletely to some of the above drugs and taking into account their potential side effects, there is still a need for additional, orally available and safe drugs with novel mechanism/s of action. We therefore examined the effect of edelfosine on human CD4^+^ T cells with the goal to assess whether this “old” compound might be useful as an immunomodulatory treatment in MS.

## Results

### Edelfosine induces cell death in a concentration-dependent manner in CD4^+^ and CD8^+^ T cells

**Figure 1:**
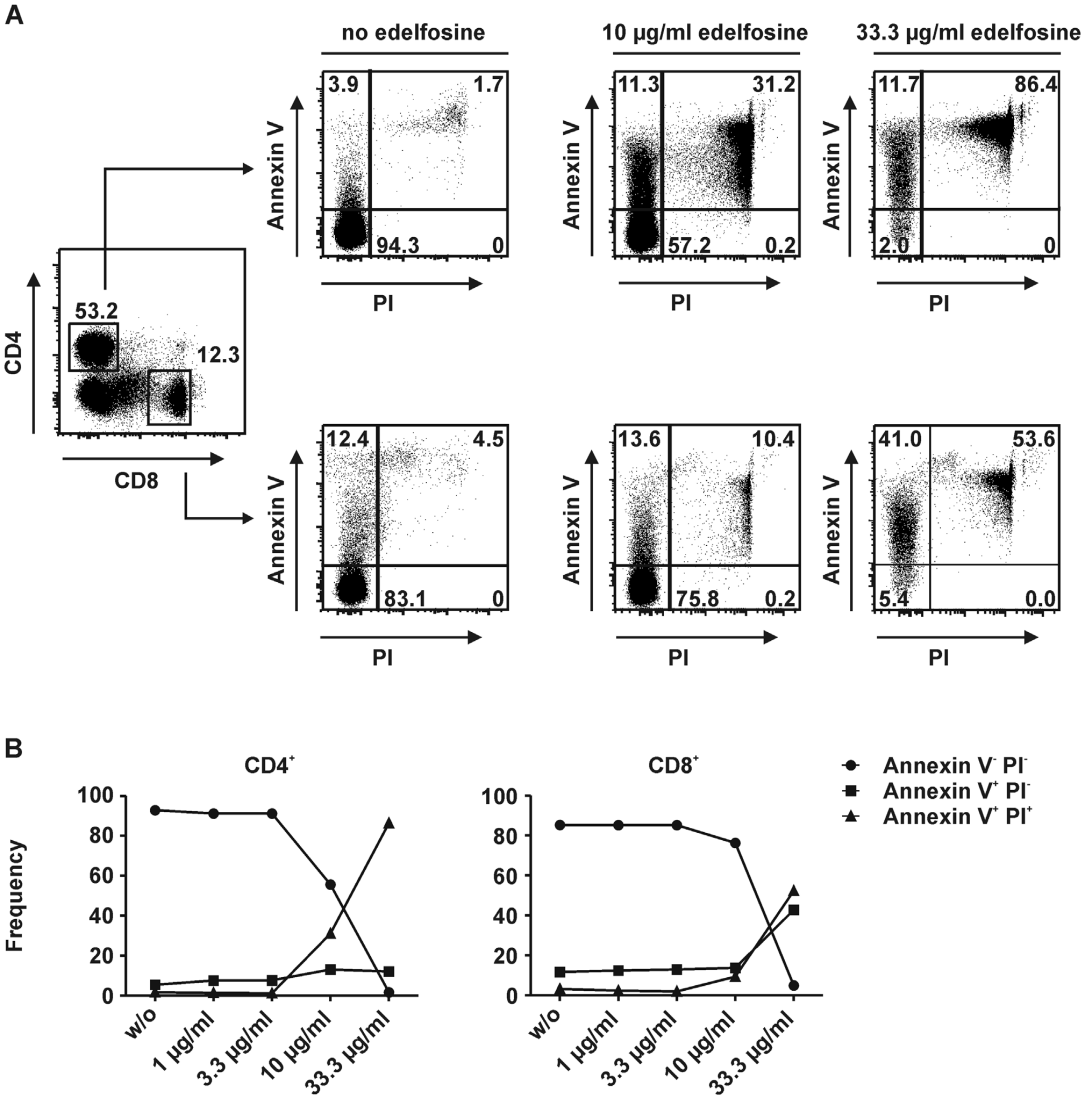
Edelfosine impact on T cell viability. (A) Gating strategy to determine frequencies of annexin V^+^ and/or PI^+^ CD4^+^ as well as CD8^+^ T cells. Dot plots for annexin V and PI gating of approaches in absence of edelfosine, with 10 µg/ml edelfosine, or 33.3 µg/ml edelfosine. (B) With regard to CD4^+^ T cells just 10 µg/ml edelfosine led to a decrease in annexin V^−^PI^−^ frequencies accompanied by a remarkable increase in annexin V^+^PI^+^CD4^+^ T cell frequencies. In the case of CD8^+^ T cells a marked decrease in annexin V^−^PI^−^ cells was only observed with 33.3 µg/ml edelfosine which resulted in comparable frequencies of annexin V^+^PI^−^ as well as annexin V^+^PI^+^ cells. PBMCs of a donor were seeded in triplicates and pooled before analysis.

To examine the effect of various edelfosine concentrations on human T cells and to determine a possible damaging range of concentrations, peripheral blood mononuclear cells (PBMCs) were isolated from human blood and incubated for 24 h in presence of various edelfosine concentrations. After gating on CD4^+^ and CD8^+^ populations apoptotic cells and dead cells were analyzed by examining annexin V^+^ and/or propidium iodide (PI)^+^ cell populations (Fig. 1A). The incubation of PBMCs with 10 µg/ml edelfosine led to a decrease of the annexin V^−^PI^−^ frequencies in CD4^+^ T cells compared to frequencies without edelfosine, with 1 µg/ml or 3.3 µg/ml edelfosine (Fig. 1B). The incubation in the presence of 33.3 µg/ml edelfosine resulted in only 2.0% annexin V^−^PI^−^CD4^+^ T cells, i.e. only a minority of cells did not show signs of apoptosis/cell death. Correspondingly, the frequencies of annexin V^+^PI^+^CD4^+^ T cells increased reaching a maximum after culture in the presence of 33.3 µg/ml edelfosine. Here, the frequencies of annexin V^+^PI^−^CD4^+^ T cells showed only a mild increase with increasing edelfosine concentrations. Similarly, annexin V^+^PI^−^CD8^+^ and annexin V^+^PI^+^CD8^+^ populations displayed a comparable increase in frequencies with increasing edelfosine concentration. Thus, 33.3 µg/ml edelfosine appeared to interfere massively with cell viability. Frequencies remained unchanged upon culture without edelfosine and also with 1 µg/ml, 3.3 µg/ml or 10 µg/ml edelfosine. By affecting cellular viability in unstimulated T cells, at higher concentrations edelfosine may also interfere with the cells' functional capacity to respond to antigen and to proliferate. This introductory experiment confirmed previous seminal findings which state that an apoptotic response is optimally induced *in vitro* after treatment with 3–5 µg/ml edelfosine [23], e.g. Cabaner *et al.* applied 5 µg edelfosine to study apoptosis induction in PBLs cultured at 1×10^6^ cells/ml cell culture medium [4], and several other groups describe apoptosis induction in the same dose range [24], [25].

### Edelfosine interferes with proliferation of human PBMCs and of antigen-specific T cell lines

**Figure 2:**
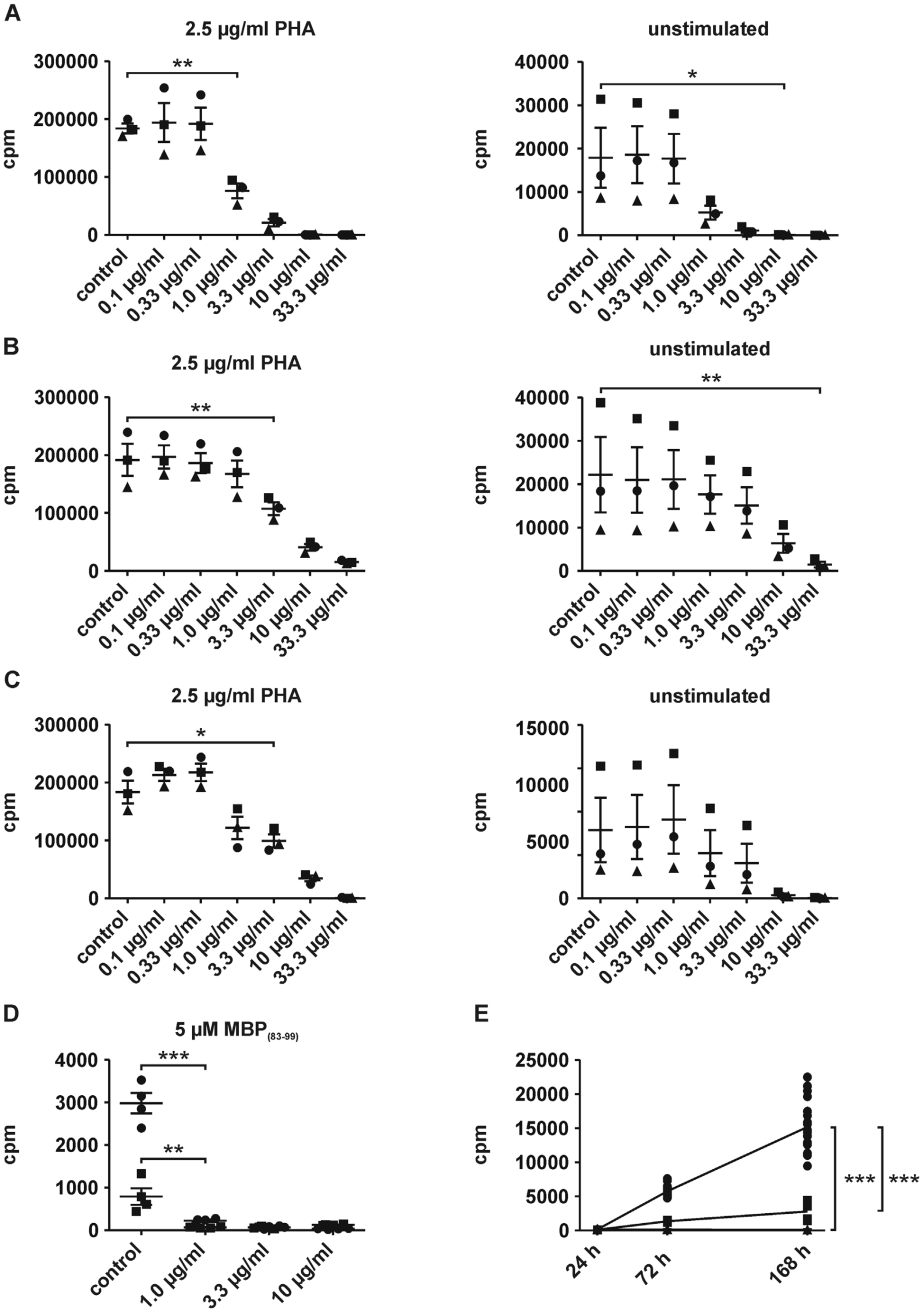
Human T cell proliferation was affected by edelfosine. (A) Reduced PBMC proliferation upon addition of edelfosine on cell seeding was independent of the addition of PHA. Notably, PHA-activated cells appeared to be susceptible to edelfosine at 10-fold lower concentrations. (B) The inhibitory effect of edelfosine was observed if the drug was added to already activated, proliferating T cells, i.e. two days after cell seeding and PHA addition. Here, a significant reduction of proliferation in unstimulated cells was only detectable with 33.3 µg/ml edelfosine. (C) Preincubation of PBMCs with at least 3.3 µg/ml edelfosine interfered with the cells' capacity to proliferate upon PHA stimulation. No effect was detected in preconditioned, but unstimulated cells (experiments A, B, C: sample size n = 3 donors, each approach was seeded in triplicates and means for each donor are represented by symbols •, ▪, ▴). (D) 1 µg/ml edelfosine or higher concentrations profoundly diminished proliferation in MBP_(83–99)_-specific TCLs. One representative TCL of two is shown. Cells were incubated in quadruplicates. • stimulated, ▪ unstimulated (E) PBMCs of one donor were cultured without addition of a stimulus. Proliferation was detectable after seven days. The presence of anti-HLA-DR- and anti-MHC class I-blocking antibodies or 3.3 µg/ml edelfosine inhibited cellular proliferation (• untreated, ▪ blocking antibodies added, ▴ 3.3 µg/ml edelfosine-treated). Bars represent mean values ± SEM, *P<0.05, **P<0.01 and ***P<0.001 after repeated measures ANOVA succeeded by Bonferroni post-hoc analysis.

As we aimed at investigating functions of edelfosine with respect to its application as an oral drug in MS and other autoimmune diseases, we examined the drug's effect on homeostatic proliferation. Our recent observation that MS patients show increased homeostatic proliferation underscores the potential involvement of this physiological process in autoimmune conditions [18]. In order to characterize the influence of edelfosine on the proliferation of human T cells, PBMCs were first stimulated with a mitogenic stimulus, PHA. We first examined the effect of edelfosine on T cell activation and proliferation by adding edelfosine immediately when seeding the cells (Fig. 2A). A reduction in proliferation was already detectable upon addition of 1.0 µg/ml edelfosine and also found at higher concentrations. Interestingly, unstimulated controls, i.e. cells seeded without antigenic or mitogenic stimulus also showed a reduction in cellular proliferation at concentrations of 1 µg/ml edelfosine or higher. The second approach aimed at characterizing the influence on already proliferating cells (Fig. 2B). Here, cells were activated with PHA, and edelfosine was added two days later. Concentrations of 3.3 µg/ml edelfosine or higher reduced proliferation. Since edelfosine affects the viability of unstimulated cells at higher concentrations, it may also compromise the capacity of T cells to proliferate upon stimulation. To address this issue PBMCs were incubated with or without edelfosine for 24 h. Prior to stimulation with PHA or only adding media, cells were washed extensively to remove edelfosine followed by further culturing for three days (Fig. 2C). In control approaches cells were incubated without PHA. Cells were found to retain their proliferative function after incubation with edelfosine at concentrations of up to 1 µg/ml. 3.3 µg/ml edelfosine or higher interfered with the cellular capacity to proliferate upon stimulation with PHA. With regard to unstimulated cells, no significant reductions of proliferation were identified following pre-incubation with edelfosine and subsequent washout. These results are in agreement with our introductory findings on the induction of cell death by edelfosine (Fig. 1) and with previous data [6], [23]. To investigate further the influence of edelfosine on T cell proliferation in the context of antigen-specific stimulation, T cell lines (TCLs) specific for MBP_(83–99)_ were used. Edelfosine was added immediately after seeding of cells (Fig. 2D). After three days of culture 1.0 µg/ml edelfosine already profoundly reduced T cell proliferation in stimulated as well as unstimulated conditions. Additionally, PBMCs were incubated for up to seven days without the addition of a stimulus (Fig. 2E). Cells were cultured in medium alone, in presence of anti-HLA-DR- and anti-MHC class I-blocking antibody or with 3.3 µg/ml edelfosine. Both the addition of the antibodies and edelfosine resulted in considerably lower proliferation. In the case of edelfosine the proliferation was reduced to baseline levels (Fig. 2E, Supplementary Fig. S1). These results indicate an effect of edelfosine on cellular proliferation even if cells were not activated by adding a defined stimulus, i.e. in the unstimulated condition or homeostatic proliferation setting.

### Transcriptome analysis of CD4^+^ T cells identifies novel mechanisms of action of edelfosine

We next performed genome-wide expression studies with human CD4^+^ T cells to characterize, which mechanisms were involved in the above activities of edelfosine on T cells, and whether it exerts as yet unknown, additional effects. Unstimulated human CD4^+^ T cells were exposed to two concentrations of edelfosine (low: 3.3 and high: 10 µg/ml) and stimulated CD4^+^ T cells only to the low concentration. Edelfosine modulated the gene expression both in the stimulated and unstimulated condition, although to a more limited extent in the latter (Table 1). Except for the TCF4 gene, every gene that was significantly downregulated at the low edelfosine concentration in unstimulated CD4^+^ T cells was also observed at the high one. At the high dose 11 genes were up- (signal log ratio (SLR) ≥0.8) and 60 downregulated (SLR ≤−0.8). Therefore, we focused for further analysis of unstimulated cells on the high edelfosine dose. Subsequently, the database for annotation, visualization and integrated discovery (DAVID) was used to annotate the differentially expressed genes to functional themes/groups and assign to them gene-ontology (GO) terms for specific pathways of cellular function [26]. Tables 2A and -B summarize the top-3 GO-terms, which were selected due to their P-values. As expected from previously known mechanisms, edelfosine upregulated genes associated with apoptosis and cell death in unstimulated CD4^+^ T cells, which is consistent with the above detection of annexin V and PI after edelfosine incubation (Table 2A). A novel and interesting finding was the downmodulation of MHC class II-associated genes and genes related to antigen processing and presentation (Table 2A). In stimulated CD4^+^ T cells edelfosine downregulated genes involved in cell cycle progression and upregulated apoptosis-related genes. Again, a set of unexpected genes that were upregulated by edelfosine has been identified. These genes are involved in immune responses in general and responses to virus. Interestingly, most of these genes were found to be type I IFN-associated genes (Table 2B).

**Table 1:** Modulated gene expression in CD4^+^ T cells upon culture with edelfosine.

Pairwise comparison	SLR ≤−0.8	SLR ≥0.8
*Unstimulated*		
3.3 µg/ml edelfosine vs. no edelfosine	21	0
10 µg/ml edelfosine vs. no edelfosine	60	11
*Stimulated*		
3.3 µg/ml edelfosine vs. no edelfosine	665	288

Numbers of differentially up- or downregulated genes after incubation with edelfosine are shown.

**Table 2:** Clustering of up- or downregulated genes to determine biological pathways affected by edelfosine in human CD4^+^ T cells after gene expression analysis.

A Setting: unstimultated + 10 µg/ml edelfosine vs. unstimulated		
Up	Gene Examples	P-value
Apoptosis	JUN, RHOB, KRAS	1.8×10^−2^
Programmed cell death	JUN, RHOB, KRAS	1.9×10^−2^
Learning	JUN, KRAS	2.2×10^−2^
Down	Gene Examples	P-value
Immune response	CD74, CD79A, IGJ, IRF8, CCL22	3.9×10^−14^
Antigen processing and presentation of peptide or polysaccharide antigen via MHC class II	CD74, IFI30, HLA-DMA, HLA-DPA1	2.2×10^−11^
Antigen processing and presentation	CD74, IFI30, HLA-DMB, HLA-DRA	1.3×10^−8^

B Setting: stimulated + 3.3 µg/ml edelfosine vs. stimulated		
Up	Gene Examples	P-value
Immune response	IFIH1, IFI6, IFI44L, TNFSF10, CXCR4	2.6×10^−10^
Response to virus	IRF7, ISG20, IFI44, MX1, MX2, RSAD2	7.5×10^−10^
Regulation of apoptosis	CASP10, CARD16, DDIT3, HSPA1A	1.0×10^−8^
Down	Gene Examples	P-value
Cell cycle	BARD1, CKS1B, E2F1, CDCA2, CCNF	3.4×10^−81^
Cell cycle phase	CKS2, DMC1, E2F1, RAD51, CCNA2	2.6×10^−80^
M phase	FBXO43, MAD2L1, MKI67, CENPV	3.0×10^−77^

(A) The incubation of unstimulated cells with 10 µg/ml edelfosine resulted in the upregulation of apoptosis- and cell death-associated genes. Genes involved in immune response and antigen processing and presentation were downregulated. (B) In the case of stimulated cells which were cultured in presence of 3.3 µg/ml edelfosine the downmodulation of cell cycle progression-related genes was found. Additionally, edelfosine resulted in the upregulation of genes assigned to immune response- and virus response-pathways characterized by type I IFN-regulated genes.

We then focused on the genes related to the newly identified biological pathways that are modulated by edelfosine. Supplementary Table S1A lists the genes related to MHC class II, and antigen processing and immunoglobulin/B cell regulation that were significantly downregulated in unstimulated cells upon exposure to edelfosine. The second gene list depicts genes that were upregulated by stimulated CD4^+^ T cells in the presence of edelfosine (Supplementary Table S1B) and which have been validated by real-time RT-PCR for IFIT1, IFIT2, IFIT3 and IFI44 (Supplementary Fig. S2). Among the genes that are known to be regulated by type I IFN are some that were previously detected in a longitudinal gene expression study of PBMCs derived from IFN-β-treated MS patients [27].

Supplementary Figures S3A and –B attempt to visualize the effects of edelfosine by creating heatmaps. The first heatmap focuses on the downregulated genes related to MHC class II, antigen processing and –presentation, and immunoglobulin/B cell regulation (Supplementary Fig. S3A). The heatmap indicates the consecutive downregulation of genes with increasing edelfosine concentration. Only genes with at least one SLR ≤−0.8 in all three possible pairwise comparisons are shown. The second heatmap shows upregulated genes related to immune response and anti-viral response by comparing the “unstimulated” condition to the conditions “stimulated” and “stimulated in the presence of 3.3 µg/ml edelfosine” (Supplementary Fig. S3B). The heatmap emphasizes the consistent upregulation of immune and virus response-associated, type I IFN-related genes upon stimulation in presence of 3.3 µg/ml edelfosine. In summary, edelfosine treatment of unstimulated T cells led to the downregulation of MHC class II genes and a type I IFN signature could be identified in stimulated T cells. Both effects of edelfosine may alone or jointly be responsible for the reduced proliferation.

### Edelfosine-induced downmodulation of MHC class II surface expression of B cell subsets

**Figure 3:**
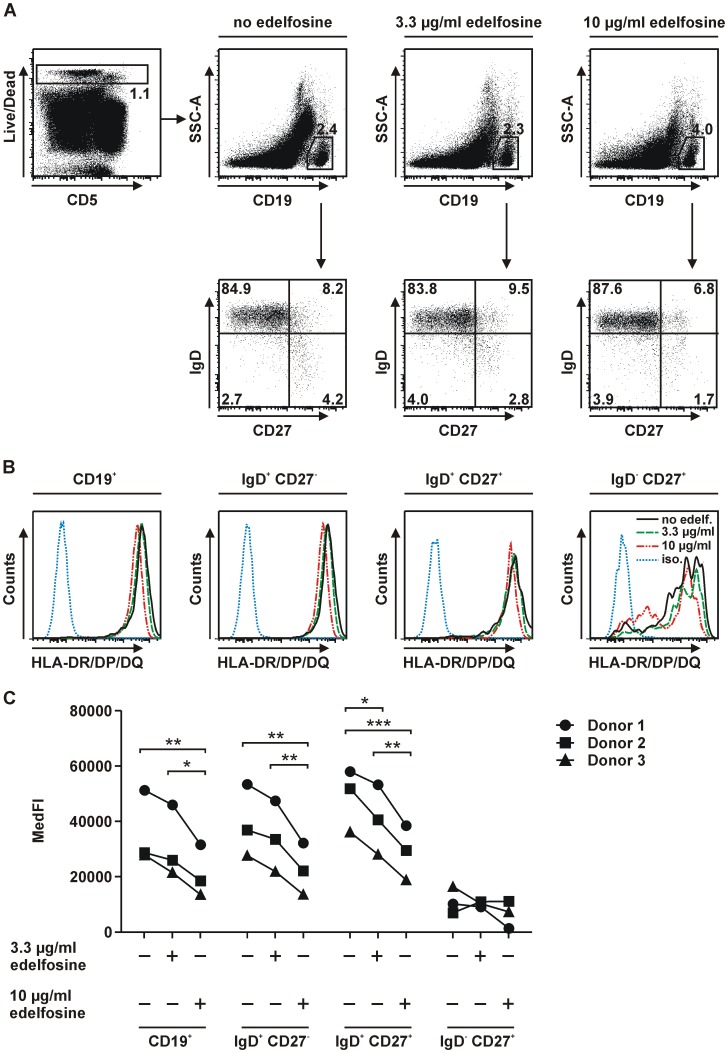
Edelfosine impact on HLA-DR/DP/DQ expression on human B cells. (A) Gating strategy to identify viable CD19^+^ B cells and their IgD^+^CD27^−^, IgD^+^CD27^+^ and IgD^−^ CD27^+^ subsets after the incubation of PBMCs for 24 h in the absence of edelfosine or in the presence of 3.3 µg/ml and 10 µg/ml edelfosine, respectively. (B) Histograms, exemplary of one donor, display the edelfosine-induced downmodulation of HLA-DR/DP/DQ on the previously described B cell subsets in comparison to the respective untreated control approach. (C) Summary of MedFI values determined for each treatment within each B cell subset (n = 3 donors (represented by symbols •, ▪, ▴), + edelfosine added as indicated, − no edelfosine added). For CD19^+^, IgD^+^CD27^−^ and IgD^+^CD27^+^ populations significant reductions of HLA-DR/DP/DQ expression were observed (*P<0.05, **P<0.01, ***P<0.001 after repeated measures ANOVA and Bonferroni post-hoc analysis). Histogram legends for B: no edelfosine (black line), 3.3 µg/ml edelfosine (green line), 10 µg/ml edelfosine (red line), isotype control (blue line).

**Figure 4:**
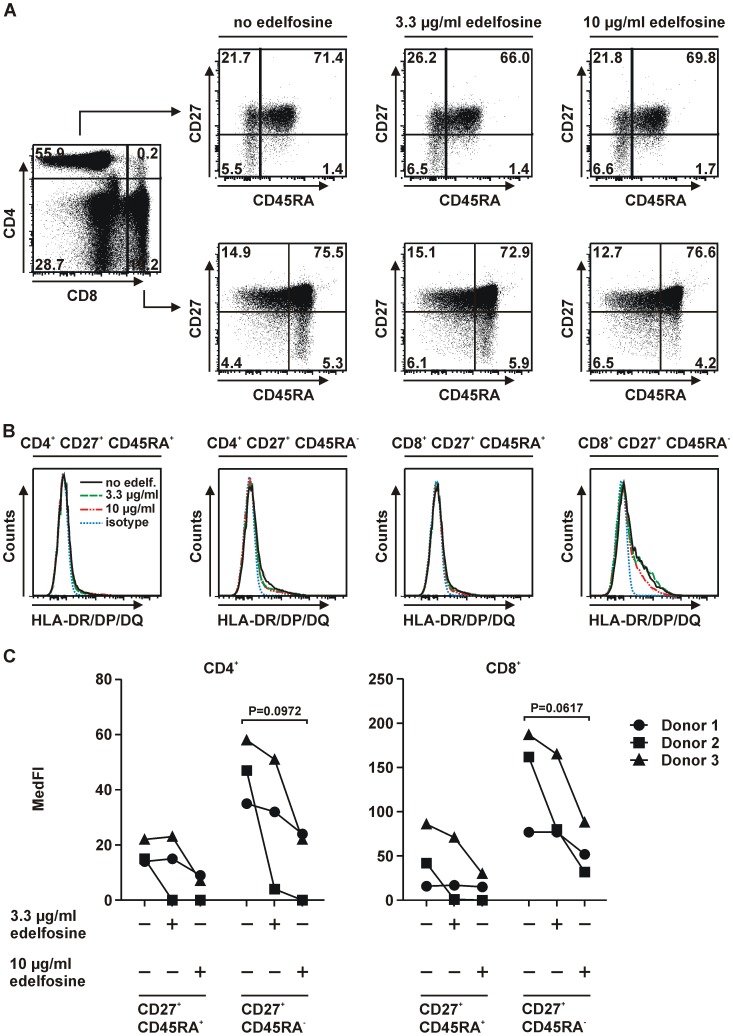
Edelfosine impact on HLA-DR/DP/DQ expression on human T cells. (A) Gating strategy to identify viable CD4^+^ and CD8^+^ T cells and their naïve (CD27^+^CD45RA^+^) and memory (CD27^+^CD45RA^−^) subsets after the incubation of PBMCs for 24 h in the absence of edelfosine or in the presence of 3.3 µg/ml and 10 µg/ml edelfosine, respectively. (B) Representative histograms of one donor display the considerably low expression of HLA-DR/DP/DQ on the previously described T cell subsets. (C) Summary of MedFI values determined for each treatment within each T cell subset (n = 3 donors (represented by symbols •, ▪, ▴), + edelfosine added as indicated, − no edelfosine added). For CD27^+^CD45RA^+^ and CD27^+^CD45RA^−^ populations of CD4^+^ and CD8^+^ T cells no significant reduction of HLA-DR/DP/DQ expression was observed after Bonferroni post-hoc analysis (depicted P-value: as determined by repeated measures ANOVA). Histogram legends for B: no edelfosine (black line), 3.3 µg/ml edelfosine (green line), 10 µg/ml edelfosine (red line), isotype control (blue line).

The microarray studies of CD4^+^ T cells revealed that resting, i.e. unstimulated cells downregulated the expression of genes associated with MHC class II antigen presentation in the presence of edelfosine. Since CD4^+^ T cells are not considered “classical” antigen-presenting cells (APCs), we therefore examined HLA-DR/DP/DQ expression in CD4^+^ and CD8^+^ T cells and additionally in B cells as classical APCs. PBMCs were incubated for 24 h without edelfosine, in presence of 3.3 µg/ml or 10 µg/ml edelfosine. After excluding dead cells CD19^+^ B cells were selected (Fig. 3A). The further subdivision by the surface markers IgD and CD27 allowed to define the effect of edelfosine on MHC class II expression of distinct B cell populations. Adult peripheral blood B lymphocytes can be divided by these markers into discrete subsets with increasing differentiation: IgD^+^CD27^−^, IgD^+^CD27^+^ and IgD^−^CD27^+^ B cells. The median fluorescence intensities (MedFIs) for HLA-DR/DP/DQ expression on B cell subsets were determined by flow cytometry (Fig. 3B). With regard to CD19^+^ B cells en bloc, but also for IgD^+^CD27^−^ and IgD^+^CD27^+^ B cell subsets the culture with edelfosine resulted in a concentration-dependent downregulation of HLA-DR/DP/DQ expression on the cell surface (Fig. 3C, Supplementary Table S2A). In comparison, the expression levels appeared to be rather low on IgD^−^CD27^+^ memory B cells. In the latter population no differences in HLA-DR/DP/DQ expression levels were found. Since edelfosine was initially found to affect MHC class II-related genes in CD4^+^ T cells the impact of edelfosine on HLA-DR/DP/DQ expression of T cells was also assessed. After PBMC isolation followed by incubation for 24 h, cells were stained to gate on CD4^+^ and CD8^+^ T cells preceded by the exclusion of dead cells (Fig. 4A). The MedFIs for HLA-DR/DP/DQ expression on naïve and memory CD4^+^ and CD8^+^ T cells were determined (Fig. 4B). Memory T cells, which showed an approximately 2-fold higher HLA-DR/DP/DQ expression compared to naïve T cells, pointed to a comparable tendency in reduced HLA class II expression as found in B cells (Fig. 4C, Supplementary Table S2B). Hence, in the case of B cells as classical HLA-DR/DP/DQ-expressing APCs the impact of edelfosine on MHC class II expression previously identified by microarray analysis was verified.

### Edelfosine reduces IFN-γ secretion of stimulated CD4^+^ T cells

**Figure 5:**
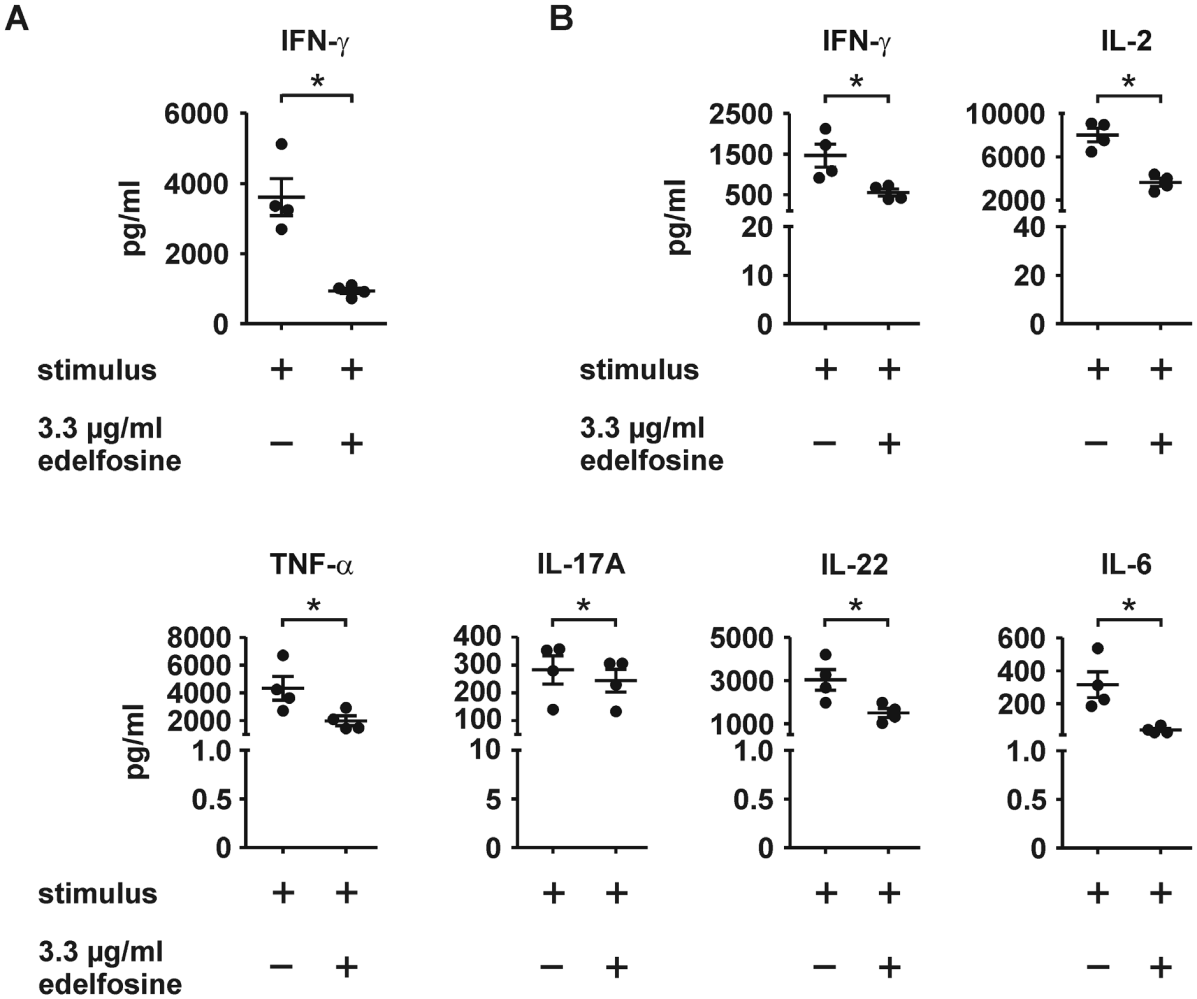
Cytokine secretion was modulated in activated CD4^+^ T cells by edelfosine. (A) By ELISA, a significant reduction of IFN-γ-secretion was monitored upon edelfosine treatment. (B) This result was confirmed by a human 13plex kit which allowed the detection of not only reduced concentrations of IFN-γ in supernatants of edelfosine-treated cells, but also reduced concentrations of the Th1-associated cytokines IL-2 and TNF-α as well as the Th17-associated cytokines IL-17A, IL-22 and IL-6. Cells of four individuals (age-matched, two males and two females) were used, error bars indicate SEM of respective means (*P<0.05 after paired t-tests).

Whole genome expression analysis of activated, edelfosine-treated CD4^+^ T cells indicated the upregulation of genes with close association to immune response and response to virus. To exclude that this upregulation of type I IFN-associated genes was due to the presence of IFN-α or -β in the cell culture medium, cell culture supernatants were analyzed by ELISA to determine the concentrations of IFN-α and IFN-β, but also IFN-γ. IFN-α as well as IFN-β were virtually undetectable by ELISA in supernatants of stimulated CD4^+^ T cells after 30 h of incubation in both absence or presence of 3.3 µg/ml edelfosine. Instead, supernatants of cells cultured without edelfosine contained 3,606.09±525.12 pg/ml IFN-γ. 3.3 µg/ml edelfosine reduced IFN-γ levels to 940.78±81.23 pg/ml, which is equivalent to a −3.83-fold reduction (Fig. 5A). The downmodulation of IFN-γ was verified by using the flow cytometry-based human Th1/Th2/Th9/Th17/Th22 13plex Kit FlowCytomix (Fig. 5B). Edelfosine led to an IFN-γ-reduction from 1,462.76±282.26 pg/ml to 552.93±86.61 pg/ml (−2.65-fold less). Moreover, the Th1-related cytokines IL-2 and TNF-α were also reduced in comparison to their respective controls in absence of edelfosine. Furthermore, Th17-associated cytokines IL-17A, IL-22, as well as IL-6 were reduced by edelfosine.

## Discussion

Despite enormous progress in the development of MS treatments there remains a need for therapies that combine high efficacy with oral availability, that have a novel mechanism of action and at the same time exhibit few and only minor side effects. Increased autologous/homeostatic proliferation in the context of HLA-DR15 in MS [18] probably influences the composition of the immune repertoire and might contribute to MS pathogenesis at several levels. Together with the observation that homeostatic proliferation contributes to secondary autoimmune diseases following treatment of MS patients with alemtuzumab [28], it provides an interesting rationale for new treatments of MS. Based on the data presented here, we assume that the synthetic ether lipid edelfosine, which efficiently blocks homeostatic proliferation, appears an interesting option. In addition to its documented benign safety profile in humans [25], [29] and oral availability edelfosine shows interesting and novel mechanisms of action, which are discussed below.

Regarding its effects on T cells the concentration of edelfosine determines whether it induces apoptosis, which is well documented by prior data [6], or preferentially prevents cell proliferation as we demonstrate here. Due to its chemical structure edelfosine inserts into the lipid bilayer of plasma membranes, where it integrates into lipid rafts thereby recruiting Fas/CD95, FADD and caspase-8 [6]. These DISC-raft clusters are implicated to elicit FasL-independent apoptosis, and the number of drug molecules per cell, the cell density/dilution and the cell types are important determinants of the drug's cellular toxicity at higher concentrations [30]. In our study edelfosine also interfered with the activation and proliferation of mitogen-stimulated PBMCs in a dose-dependent manner. Results are in agreement with the data on apoptosis induction and cell death since the frequencies of viable CD4^+^ T cells were reduced at concentrations of 10 µg/ml edelfosine or higher, while lower concentrations did not affect cell viability, but effectively interfered with T cell proliferation after preincubation and also when added during or 48 h after T cell activation.

These effects were observed with mitogen-activated cells and also with antigen-specific T cells, i.e. myelin-specific TCLs. Our data not only show the inhibition of proliferation of activated cells by edelfosine, but also of cells that have not been stimulated by the addition of peptide MBP_(83–99)_. Hence, edelfosine interferes with proliferation following full activation of T cells with either mitogen or specific antigen, but also with the “background”-proliferation in the unstimulated control condition. The latter proliferation may reflect at least in part a process named “homeostatic proliferation”. Homeostatic proliferation depends on contact to self peptide-MHC ligands and cytokines (e.g. IL-7) provided by other cells, e.g. classical APCs and probably T cells, in order to prevent apoptosis of T cells that have not been activated by their cognate antigen [31], [32]. Such interactions are important for thymic selection and peripheral maintenance [33], [34]. Also, both animal experiments and data with human myelin-specific T cell clones have shown that contact between fully activated dendritic cells and T cells may induce distinct activation (induction of IL-12Rβ_2_ and IFN-γ expression, Ca^2+^ currents) and long term survival of T cells upon cell-cell contact [35], [36]. Besides its physiological roles, our [18] and other investigators' recent data [28] indicate that homeostatic proliferation is likely involved in maintaining and/or expanding autoreactive T cells during MS with the result of premature senescence of T cells and narrowing of the T cell repertoire [37] and probably also a factor that contributes to increased secondary autoimmunity following treatment with the depleting anti-CD52 antibody alemtuzumab [28]. Based on these considerations the inhibition of T cell proliferation at lower concentrations of edelfosine that do not induce apoptosis is of interest, although extrapolations from our *in vitro* experiments to the *in vivo* situation in patients need to be done with caution.

In order to develop a better understanding, which mechanisms contribute to the above *in vitro* effects of edelfosine, we performed gene expression profiling studies with purified human CD4^+^ T cells. These confirmed known mechanisms of edelfosine such as apoptosis induction and cell cycle arrest, but also demonstrated previously unknown activities that may explain the above effects on T cell proliferation. At non-toxic concentrations (3.3 µg/ml) edelfosine interferes with CD4^+^ T cell proliferation of activated T cells and after preincubation, and also inhibits homeostatic T cell proliferation. Based on these data and the induction of apoptosis/cell death at 10 µg/ml edelfosine, gene expression profiling was performed with 3.3 µg/ml or 10 µg/ml edelfosine. At both concentrations a clear and unexpected downmodulation of genes assigned to immune response and antigen processing and presentation via MHC class II was observed (lowest GO P-value: 3.9×10^−14^ at 10 µg/ml edelfosine). This result is of interest in several respects. MHC class II on professional and non-professional APCs (DCs, B cells, macrophages, thymic epithelial cells) serve as recognition structures that present antigenic peptides to the T cell receptor (TCR) of CD4^+^ T cells and hence are central for physiological immune responses against pathogens, but also in the context of autoimmune diseases like MS. In MS, downmodulation of HLA class II or molecules involved in class II-associated processing by edelfosine may therefore affect the processing and presentation of autoantigens. Since interactions of autoreactive TCRs with HLA/peptide complexes are usually of lower avidity [38], we assume that these will be blocked more efficiently than responses to e.g. viral peptides. Moreover, since HLA/self-peptide complexes are besides IL-7 the strongest stimulus for homeostatic proliferation, edelfosine will probably act on this aspect *in vivo* as well as indicated by our above data. We have previously shown that B cells upregulate HLA class II during homeostatic proliferation experiments *in vitro* [18], and therefore edelfosine may influence this mechanism *in vivo* based on the reduction of HLA class II expression on B cells. Further, mouse- and human experimental data demonstrated that class II expression is involved in T cell-DC interactions in the absence of nominal antigen [35], [36]. In all these settings downmodulation of MHC class II by edelfosine can impact on both antigen-driven and homeostatic proliferation. In the present experiment CD4^+^ T cells were cultured without or with edelfosine in the absence of stimulation. IFN-γ concentrations were at baseline undetectable in both conditions (not shown), and therefore deprivation of IFN-γ upon edelfosine addition probably does not account for the observed reduced transcription of MHC class II-associated genes and also of class II surface expression of human B cells. At present we do not know the underlying molecular mechanisms, by which edelfosine interferes with transcription of molecules involved in class II-associated processing/presentation. The multiplex cytokine data indicate that edelfosine may interfere with vesicular transport of cytokines and HLA class II to the T cell surface. Hence, it may act as an immunomodulatory drug by integration into the phospholipid bilayer of transport vesicles and/or endosomal compartments for MHC class II loading. However, this remains speculation and clearly merits further study. Of particular note in the context of MS, type I IFN-associated IRF8 was downregulated and stood out against numerous type I IFN-associated genes which were upregulated when T cells were stimulated and edelfosine-treated (discussed later). IRF8 is involved in microgliogenesis [39]. Microglia are considered particularly important during chronic phases of MS, and three MS-associated SNPs within the IRF8 gene have been described [13], [40]. Therefore, downmodulation of IRF8 may be an additional interesting effect when considering edelfosine as MS treatment.

When activated CD4^+^ T cells were exposed to edelfosine it induced the downregulation of genes involved in cell cycle progression. This effect of edelfosine has been examined in previous reports. Interestingly, edelfosine inhibits cell division, but it does not interfere with nuclear division, and therefore multinucleated cells accumulate in G2/M phase of the cell cycle followed by apoptosis [41]. Thus, edelfosine is able to directly induce apoptosis by DISC complex formation, but also by indirect effects via inhibition of cytokinesis. The relative contribution of each depends on the cell type as well as the drug dose [42]. Besides blocking the biosynthesis of PC [7], ALPs can also interfere with PC breakdown into phosphatidic acid (PA) by phospholipase D and its subsequent degradation to diacylglycerol (DAG) [43]. PA and DAG act as lipid second messengers on the MAPK pathway, for instance the Ras/Raf/MEK/ERK pathway of cell proliferation. In this regard, the biosynthesis and turnover of phospholipids is not only important for the maintenance of membrane integrity, but also serves as a stock for precursors of lipid second messengers that mediate cellular function, survival, and proliferation.

A further finding of high interest was the prominent upregulation of type I IFN-associated genes upon incubating stimulated CD4^+^ T cells with edelfosine. Type I IFNs bind to heterodimeric type I IFN receptors, which are composed of IFNAR1 and IFNAR2 subunits, which signal through phosphorylated STAT1/STAT2 into the nucleus, where they initiate transcription of IFN-α/β-inducible genes. IFN-α/β activates also other pathways, for instance the formation of STAT1 homodimers can induce the transcription of genes that contain the IFN-γ-activated sequence (GAS). Of note, cross-talk by feedback circuits have been reported for IFN-α/β and the type II IFN, IFN-γ [44], [45]. Therefore, type I and II IFNs may influence each other in terms of signaling and production. In our experiments, type I IFNs were not detectable in supernatants of CD4^+^ T cells, which indicates that the theoretical autocrine or paracrine type I IFN-induced gene expression is unlikely. Moreover, data from both ELISA and multiplex cytokine detection demonstrate that edelfosine inhibits the expression and/or secretion of IFN-γ by CD4^+^ T cells. This effect occurs despite the edelfosine-induced expression of type I IFN-associated genes. However, no downregulation of IFN-γ-associated genes was found by microarray analysis. The inhibition by edelfosine affected not only the secretion of IFN-γ but also of other cytokines and therefore is a more general effect. We therefore conclude that the detected upregulation of type I IFN-associated genes in stimulated, edelfosine-treated CD4^+^ T cells is not due to the presence of either IFN-α or –β in the cell culture supernatant.

Several studies have examined the effects of type I IFN during treatment of MS patients with IFN-β. Serrano-Fernandez *et al.* performed a longitudinal analysis over one year of gene expression in PBMCs during IFN-β-1b treatment of MS patients [27]. 14 significantly upregulated genes were identified at all three time points. These had also previously been shown to be differentially expressed upon treatment with IFN-β-1a (IFI44L, ISG15) [46], [47], IFN-β-1b (IFIT3, SN/SIGLEC1) [48] or both (EI2AK2, IFI6, IFI44, IFIH1, IFIT1, IFIT2, MX1, OASL, RSAD2 and XAF1) [46]–[50]. Further IFN-β treatment-related biomarkers like IL-8 were identified only directly after onset of therapy. The authors identified IFI44L, IFIT1 and RSAD2 to show the greatest fold-change. By incubating stimulated human CD4^+^ T cells with edelfosine, these candidates were also found to be highly upregulated (SLR: 3.3 to 3.4) and surpassed only by IFI44 (3.4) and IFIT2 (4.1). In principle, except for SN all 14 upregulated transcripts were also identified as upregulated genes in the case of edelfosine-incubated stimulated CD4^+^ T cells. Additionally, further type I IFN-regulated genes were identified. Functionally, transcripts can be grouped according to their role in immune response and/or response to virus. Stürzebecher *et al.* compared the differential gene expression of responders, initial responders, who produced neutralizing antibodies in the course of treatment, and non-responders to identify unique responder expression profiles [51]. Responders were found to upregulate nine genes including OAS but also TRAIL, potentially linking IFN-β treatment to elevated apoptosis. Furthermore, type I IFNs possess anti-proliferative properties possibly by amplifying FasL/Fas-induced apoptosis [52] and induction of p53 [53]. For instance, apoptosis mediated by IFN-β in melanoma cell lines was dependent on the cleavage and activation of caspase-3, -8 and -9, cytochrome c release from mitochondria and DNA fragmentation. Moreover, the expression of TRAIL was induced [54], and the IFN-β-inducible XAF1 may act as an intermediate regulator for TRAIL-induced apoptosis [55].

As expected, genes attributed to the regulation of apoptosis were also upregulated in our study. Stimulated, edelfosine-treated CD4^+^ T cells showed a significant upregulation of TRAIL and APRIL. The binding of TRAIL to its receptor can activate MAPK8/JNK, caspase-8 and -3 [56]. Like caspase-8, upregulated caspase-10 acts as an initiator caspase independently of caspase-8 to mediate apoptosis by Fas and TNF [57]. In this way edelfosine may induce DISC complex formation and subsequent apoptosis in T cells in the absence of FasL as has been shown before [6].

In summary, our results confirm and extend previous findings that edelfosine induces multiple effects that lead to apoptosis particularly at higher drug concentrations. We further demonstrate several novel aspects of edelfosine mechanisms of action including the downmodulation of HLA class II molecules and molecules involved in the class II-associated processing/presentation pathway, and further the upregulation of a large set of type I IFN-regulated genes. Its oral availability and very good tolerability and safety, which have already been shown in several clinical trials [25], [29], together with the above mechanisms of action add to a drug profile that is very interesting in the context of treating autoimmune diseases such as multiple sclerosis.

## Materials and Methods

### Ethics Statement

The protocol, under which the studies were pursued, was approved by the responsible local IRB, i.e. the Ethik Kommission der Ärztekammer Hamburg (Number of approved protocol: 2758). All participants signed a written informed consent prior to donating any samples, and the consent documents were part of the abovementioned, approved protocol. Consent documents are kept on file as part of the conduct of studies with human samples according to international standards and as stipulated by the responsible IRB.

### Preparation of edelfosine

For *in vitro* experiments dilutions of dissolved edelfosine (medmark) in dest H_2_O were prepared and stored at −20°C until use.

### Isolation of human PBMCs

Buffy coats were diluted with PBS (PAA) and PBMCs were separated by Ficoll (PAA) gradient centrifugation (650×g, 30 min, RT). Mononuclear cells which accumulated at the interphase were resuspended in ice-cold PBS and centrifuged (550×g, 10 min, 4°C). Cells were washed in ice-cold PBS and spun down (350×g, 5 min, 4°C). The cell number was determined by staining of cells with Türks Blue (Merck).

### Generation of human antigen-specific T cell lines

T cells specific for MBP_(83–99)_ were prepared from PBMCs of a HLA-DR15-positive MS- patient. In brief, 2×10^5^ PBMCs per well were seeded in 96-well plates (Greiner Bio-One) in complete TCL medium (1% penicillin/streptomycin (Gibco), 0.05 g/l gentamycin (Lonza), 2 mM L-glutamine (Gibco), 5% human serum (PAA) in RPMI1640 medium, GlutaMAX (Gibco)) supplemented with 10 µg/ml MBP_(83–99)_ peptide (peptides&elephants). At day 7 after seeding 20 IU/ml IL-2 were added to the cells. Subsequently, at day 12 after seeding proliferating cells were counted and plated at 2×10^5^ cells/well in 96-well plates in complete TCL medium supplemented with 10 µg/ml MBP_(83–99)_ peptide, 20 IU/ml IL-2 on 1×10^5^ autologous feeder cells irradiated at 60 Gray. Cells were incubated for another 12 days and the addition of IL-2 was repeated every 3–4 days.

### Cell culture experiments

Annexin V^+^ cells after culture with edelfosine were analyzed by seeding PBMCs in triplicate at 2×10^5^ cells/well in 96-well plates. Cells were cultured in X-Vivo 15 (Lonza) supplemented with edelfosine ranging from 1 µg/ml to 33.3 µg/ml in the absence of a stimulus. After 24 h triplicates of each approach were pooled for flow cytometry. For gene expression analysis enriched CD4^+^ T cells were obtained from buffy coats of age-matched donors (two males, two females). Cells were plated at 2×10^5^ cells/well in 96-well plates. Cells were cultured for 30 h in X-Vivo 15 medium or X-Vivo 15 medium supplemented with 3.3 µg/ml edelfosine, 10 µg/ml edelfosine, bead particles coated with antibodies against CD2, CD3, and CD28 (Miltenyi Biotec), or 3.3 µg/ml edelfosine in combination with bead particles coated with antibodies against CD2, CD3 and CD28, respectively. Bead particles were loaded and used according to the manufacturer's instructions at a ratio of 1∶2 of loaded particles/cell. Cell culture supernatant was stored at −20°C and used for ELISA and 13plex FlowCytomix kit application. To analyze the MHC class II expression of CD4^+^ T cells and B cells by flow cytometry PBMCs were seeded at 2×10^6^ cells in sterile 5 ml round bottom tubes (BD Falcon). Cells were cultured in 500 µl X-Vivo supplemented with 3.3 µg/ml or 10 µg/ml edelfosine as well as particles coated with antibodies against CD2, CD3 and CD28 as indicated for 24 h.

### [methyl-^3^H]-thymidine incorporation assay

To determine T cell proliferation by [methyl-^3^H]-thymidine incorporation, PBMCs and T cells were seeded in 96-well plates at 2×10^5^ cells/well or 2×10^4^ cells/well in the case of TCLs. PBMCs were incubated in X-Vivo 15 medium supplemented with a polyclonal stimulus (beads coated with antibodies against CD2, CD3 and CD28), mitogenic PHA (Sigma Aldrich) or with a myelin peptide, MBP_(83–99)_, plus 5-fold autologous feeder cells (PBMCs) irradiated at 35 Gray for 72 h. Only in the case of the homeostatic proliferation experiment for up to seven days (Fig. 2E) PBMCs were cultured in AIM V serum free medium (Invitrogen) containing human albumin including (in some conditions) blocking antibodies against HLA-DR (L243) and MHC class I (W6/32) at 30 µg/ml each, both provided by Hans-Georg Rammensee (Department of Immunology, Institute for Cell Biology, University of Tübingen, Tübingen, Germany). Edelfosine was added as indicated. T cell proliferation was determined by the incorporation of [methyl-^3^H]-thymidine (Hartmann Analytic) after 72 h of incubation, if not indicated otherwise. Precisely, 1 µCi [methyl-^3^H]-thymidine was added to each well 16 h before harvesting the cells. For quantification of beta particle emission the cells were harvested, washed and analyzed by using a beta counter (Wallac).

### Flow cytometry

For cell death analysis PBMCs were washed in FACS buffer. Fc-receptors were blocked with anti-IgG (Jackson IR). Cells were incubated for 15 min at RT in binding buffer containing anti-CD4-APC (RPA-T4, eBioscience) and anti-CD8-PacificBlue (DK25, Dako) antibodies for cell surface staining as well as FITC Annexin V (BD Pharmingen). To discriminate between viable cells, cells that were in early apoptosis and cells that were in late apoptosis or already dead 1 mg/ml of the vital dye PI (Sigma Aldrich) was added 5 min prior to cell acquisition by flow cytometry. To investigate the influence of edelfosine on MHC class II expression by human B and T cells, cells were stained for viability by using the LIVE/DEAD Fixable Dead Cell Stain Kit (Invitrogen). Cells were washed with PBS, blocked and stained for cell surface antigen expression as described before. Antibodies were anti-CD3-PE-Cy7 (UCHT1), anti-CD4-APC (RPA-T4), both from eBioscience, anti-CD8-PacificBlue (DK25, Dako), anti-CD5-PerCP-Cy5.5 (L17F12), anti-CD19-V450 (HIB19), anti-IgD-PE (IA6-2) and anti-HLA-DR/DP/DQ-FITC (Tu39), all from BD Biosciences, as well as anti-CD27-APC-Alexa750 (CLB-27/1) and anti-CD45RA-PE-Cy5.5 (MEM-56), both from Invitrogen. Data was acquired on an LSRII flow cytometer (BD Biosciences) and analyzed with FACSDiva (BD Biosciences) and FlowJo (Tree Star) software.

### Enrichment of human CD4^+^ T cells

Negative selection of CD4^+^ T cells was performed by using the CD4 T Lymphocyte Enrichment Set (BD IMag) according to the manufacturer's instructions. The cell number was determined by staining with Türks Blue. 2×10^5^ cells were employed to determine CD4^+^ T cell purity by flow cytometry using anti-CD3 and anti-CD4 antibodies. A purity of more than 90% was routinely achieved.

### RNA isolation, cDNA synthesis and microarray analysis

CD4^+^ T cells of each approach were pooled, counted and resuspended in TRIzol reagent (Invitrogen). For RNA isolation Pellet Paint Co-Precipitant (Merck) and chloroform (Fluka Chemika) were added. After mixing, cells were incubated for 2 min at RT followed by centrifugation (12,000×g, 15 min, 4°C). The upper phase of each approach was mixed with 500 µl isopropanol (Carl Roth). After 10 min of incubation at RT the suspensions were centrifuged (12,000×g, 10 min, 4°C). Subsequently, pellets were washed in 1 ml 75% ice-cold ethanol (Carl Roth), centrifuged (12,000×g, 5 min, 4°C) and the dried pellets were resuspended in 100 µl DEPC-treated water (Ambion). For RNA concentration and purification the RNeasy MinElute Cleanup Kit (Qiagen) was used as recommended by the manufacturer. To define the RNA concentration 1 µl per purified RNA sample was transferred on a NanoDrop spectrophotometer ND-1000 (Thermo Scientific). To determine the quality of the prepared RNA the RNA 6000 Nano Kit (Agilent) was used according to the manufacturer's instructions. RNA samples were heated at 70°C for 2 min prior to use. Subsequently, Nano Chips were loaded and analyzed by using the 2100 Bioanalyzer (Agilent). Reverse transcription of RNA to synthesize cDNA was done by using the WT Expression Kit (Ambion). cDNA fragmentation and labeling was performed by applying the GeneChip WT Terminal Labeling Kit (Affymetrix). Subsequently, probes were prepared using the GeneChip Hybridization, Wash, and Stain Kit (Affymetrix) and analyzed by microarray technology (Affymetrix GeneChip Human Gene 1.0 ST Array). The gene expression scan was performed with the GeneChip Command Console 3.0 Software (Affymetrix). The robust multi-array average (RMA) algorithm was applied for background correction, using the Expression Console 1.1 Software (Affymetrix). The samples were quantile normalized, followed by mean signal summarization. Hierarchical clustering, t-test and significance analysis of microarrays (SAM) were performed using the TIGR multi experiment viewer (MeV) 4.6.1 Software (TM4 Development Group). Microarray data is available at www.ncbi.nlm.nih.gov/geo/ (GEO accession: GSE44392).

### Real-time RT-PCR

RNA was used for QuantiTect Primer Assays (Qiagen) with QuantiFast SYBR Green RT-PCR Kit (Qiagen) according to the manufacturer's instructions. Each real-time RT-PCR reaction was performed in triplicates. The relative expression of treated versus untreated cells was normalized to GAPDH expression (comparative C_T_ method). As mentioned before, CD4^+^ T cells were derived from age-matched donors (two males, two females).

### ELISA

Stored cell culture supernatants were thawed, 12 wells per approach were selected and pooled. For ELISA analysis of IFN-α and –β as well as IFN-γ according to the manufacturer's instructions (PBL Laboratories and BioLegend, respectively), 100 µl of mixed supernatant were used. Cytokines were quantified by using a microplate reader (Biotek Instruments) to determine the absorbance at 450 nm.

### Analysis of cytokine production by flow cytometry

Saved cell culture supernatants were thawed, 10 wells per approach were selected and 20 µl per well were pooled. Cytokines secreted by CD4^+^ T cells were determined by applying the Human Th1/Th2/Th9/Th17/Th22 13plex Kit FlowCytomix (eBioscience) according to the manufacturer's instructions. Briefly, 25 µl of mixed supernatants were incubated for 2 h at RT with an equal volume of Bead Mixture and 50 µl Biotin-Conjugate Mixture. Samples were washed repeatedly with Assay Buffer (200×g, 5 min). Afterwards, samples were resuspended in 100 µl Assay Buffer and 50 µl Streptavidin-PE Solution and incubated for 1 h at RT. Again, samples were washed repeatedly in Assay Buffer (200×g, 5 min). For analysis with FACSDiva and FlowCytomix Pro 2.4 (eBioscience) software, samples were resuspended in Assay Buffer and acquired using an LSRII flow cytometer.

### Statistical analysis

For analysis of human PBMC proliferation, proliferation of MBP_(83–99)_-specific TCLs as well as expression of HLA by human B and T cells Bonferroni post-hoc tests succeeded repeated measures ANOVA. Data generated by ELISA and 13plex FlowCytomix kit were evaluated by paired t-tests, microarray data was examined by t-test analysis (adjusted P-value for significant genes: P<0.01).

## Supporting Information

Figure S1 **Homeostatic proliferation is inhibited by edeldosine.** PBMCs were derived from six donors (three males, three females), seeded in triplicates to quintuplicates and cultured without addition of a stimulus. Each symbol represents the mean value for individual donors at time points and respective treatments as indicated (• no treatment, ▪ 1 µg/ml edelfosine, ▴ 3.3 µg/ml edelfosine). Proliferation of cells was detectable after seven days, but was effectively inhibited by edelfosine. Bars represent mean values ± SEM, ***P<0.001 after repeated measures ANOVA and Bonferroni post-hoc analysis.
(TIF)

Figure S2 **Edelfosine-treated, stimulated CD4^+^ T cells show increased expression of type I IFN-regulated genes.** In order to validate microarray-derived data, gene expression was analyzed by real-time RT-PCR. RNA was isolated from stimulated CD4^+^ T cells and stimulated, 3.3 µg/ml edelfosine-treated CD4^+^ T cells after 30 h of culture. The relative expression of IFIT1, IFIT2, IFIT3, and IFI44 by edelfosine-treated CD4^+^ T cells related to untreated control CD4^+^ T cells was normalized to GAPDH expression. CD4^+^ T cells were from two male as well as two female age-matched donors. Each symbol is representative of one donor, respectively. Bars represent mean values ± SEM.
(TIF)

Figure S3 **Modulation of gene expression in human CD4^+^ T cells mediated by stimulation and edelfosine addition.** (A) The incubation of cells in absence of a stimulus resulted in an edelfosine concentration-dependent downregulation of antigen processing- and presentation-associated genes. (B) The activation of cells in presence of 3.3 µg/ml edelfosine resulted in a consistent upregulation of immune and virus response-associated genes. The values of differential gene expression changes correspond to the SLR (red for upregulation, blue for downregulation, depicted as median-centered log2-signals). Genes are clustered hierarchically in the dendrogram over the expression matrix. The height of the branches is inversely proportional to the degree of neighborhood between clusters (images generated with R statistical platform 2.12, gplots package 2.8.0). Sample size n = 4 (two male and two female age-matched donors), adjusted P-value for significant genes after t-test analysis: P<0.01.
(PDF)

Table S1 **Summary of genes related to MHC class II, antigen processing and presentation, and immunoglobulin/B cell function (A) and genes related to immune response and response to virus (B).**
(DOCX)

Table S2 **Summary of means and corresponding SEM values for B cells (A) and T cells (B).** Cell types were analyzed to determine the effect of edelfosine treatment on HLA-DR/DP/DQ expression.
(DOCX)
