# Supplementary figures and images for: The Orally Available, Synthetic Ether Lipid Edelfosine Inhibits T Cell Proliferation and Induces a Type I Interferon Response
Source: PLoS One. 2014 Mar 25;9(3):e91970. doi: 10.1371/journal.pone.0091970 (PMC3965404; doi:10.1371/journal.pone.0091970)

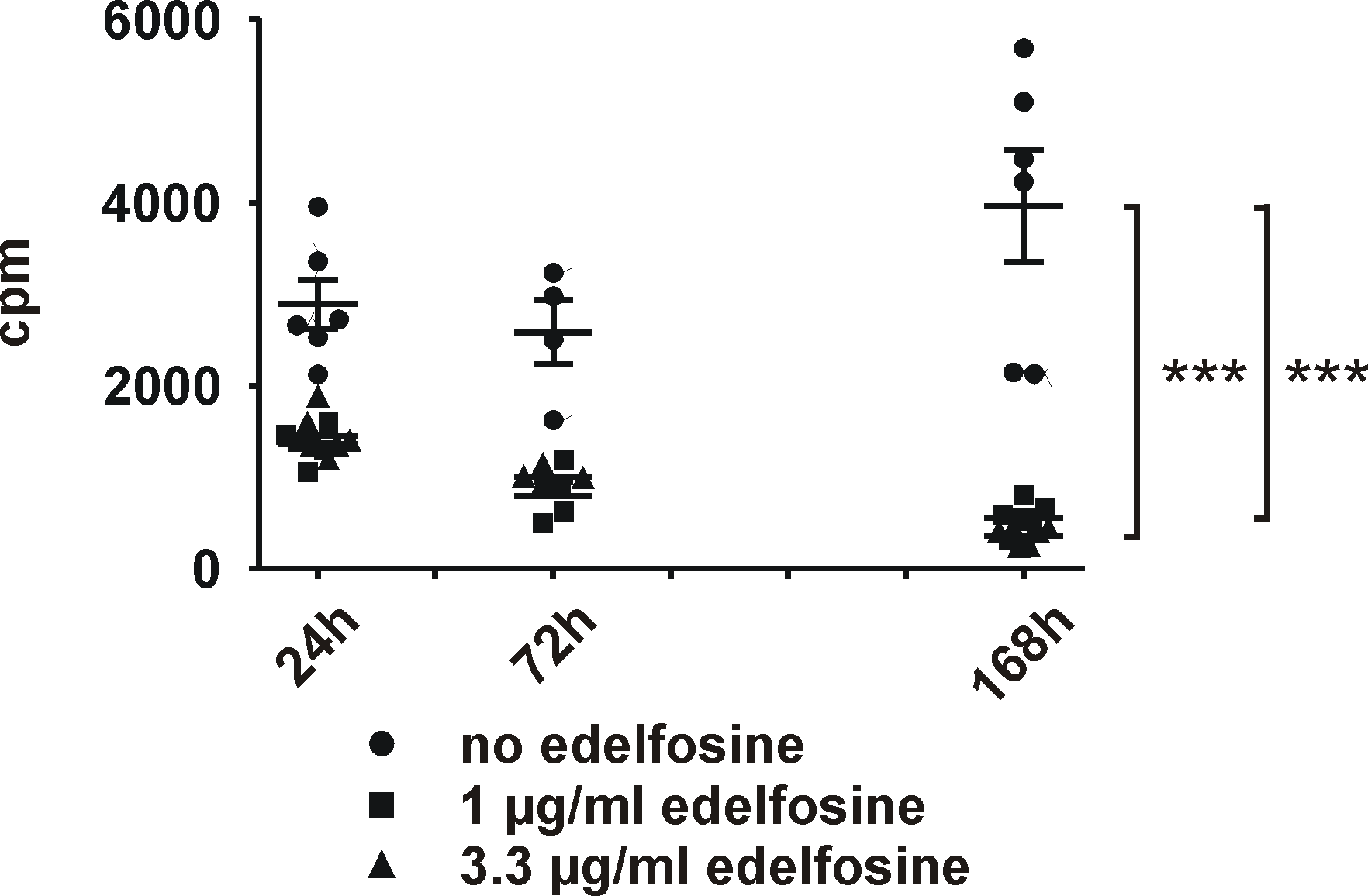

Supplement: Figure S1 — Homeostatic proliferation is inhibited by edeldosine. PBMCs were derived from six donors (three males, three females), seeded in triplicates to quintuplicates and cultured without addition of a stimulus. Each symbol represents the mean value for individual donors at time points and respective treatments as indicated (• no treatment, ▪ 1 µg/ml edelfosine, ▴ 3.3 µg/ml edelfosine). Proliferation of cells was detectable after seven days, but was effectively inhibited by edelfosine. Bars represent mean values ± SEM, ***P<0.001 after repeated measures ANOVA and Bonferroni post-hoc analysis. (TIF) [file pone.0091970.s001.tif]

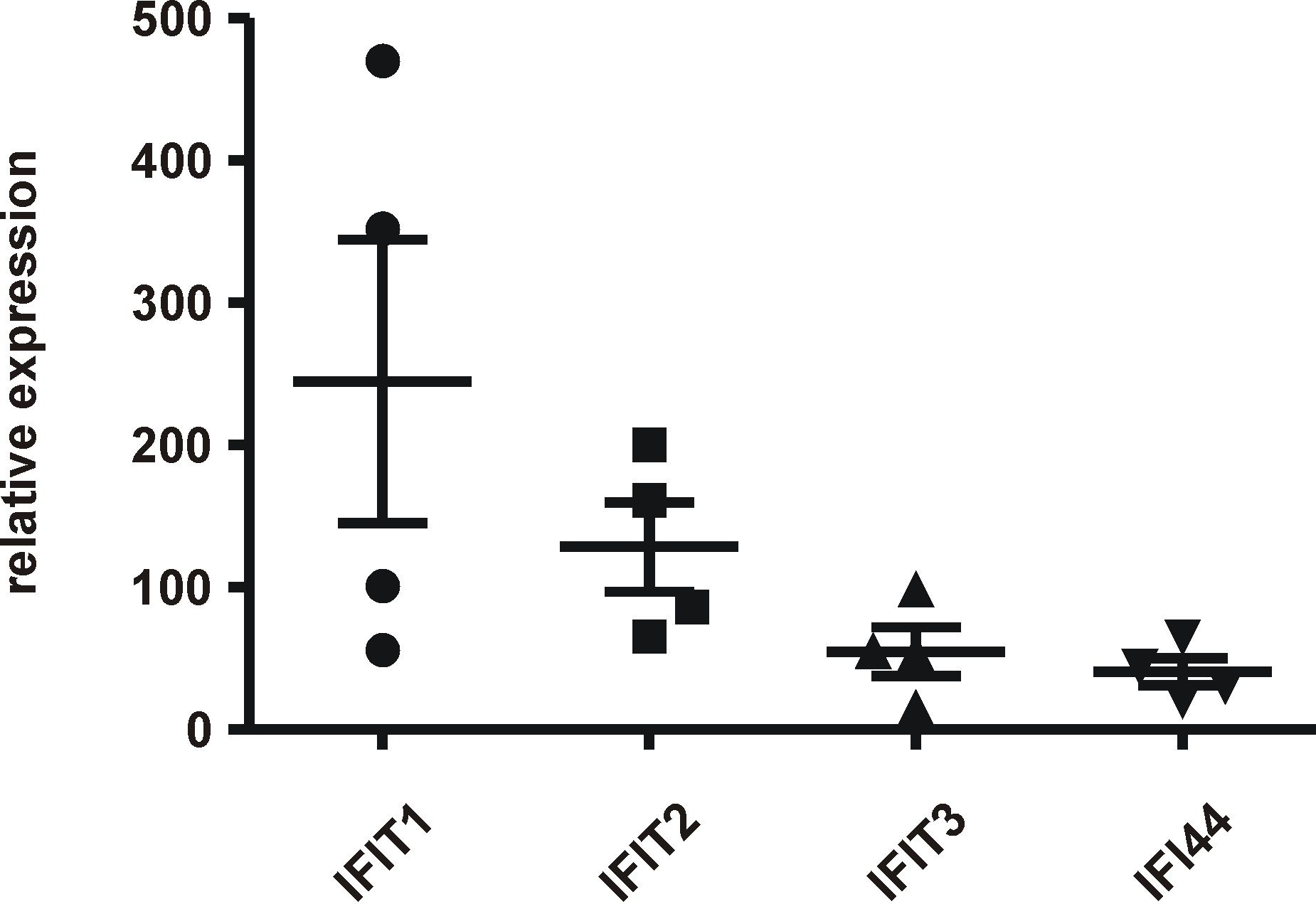

Supplement: Figure S2 — Edelfosine-treated, stimulated CD4+ T cells show increased expression of type I IFN-regulated genes. In order to validate microarray-derived data, gene expression was analyzed by real-time RT-PCR. RNA was isolated from stimulated CD4+ T cells and stimulated, 3.3 µg/ml edelfosine-treated CD4+ T cells after 30 h of culture. The relative expression of IFIT1, IFIT2, IFIT3, and IFI44 by edelfosine-treated CD4+ T cells related to untreated control CD4+ T cells was normalized to GAPDH expression. CD4+ T cells were from two male as well as two female age-matched donors. Each symbol is representative of one donor, respectively. Bars represent mean values ± SEM. (TIF) [file pone.0091970.s002.tif]
