# Supplementary material for: The Orally Available, Synthetic Ether Lipid Edelfosine Inhibits T Cell Proliferation and Induces a Type I Interferon Response
Source: PLoS One. 2014 Mar 25;9(3):e91970. doi: 10.1371/journal.pone.0091970 (PMC3965404; doi:10.1371/journal.pone.0091970)

A Edelfosine-induced downregulation of expression of genes for antigen processing

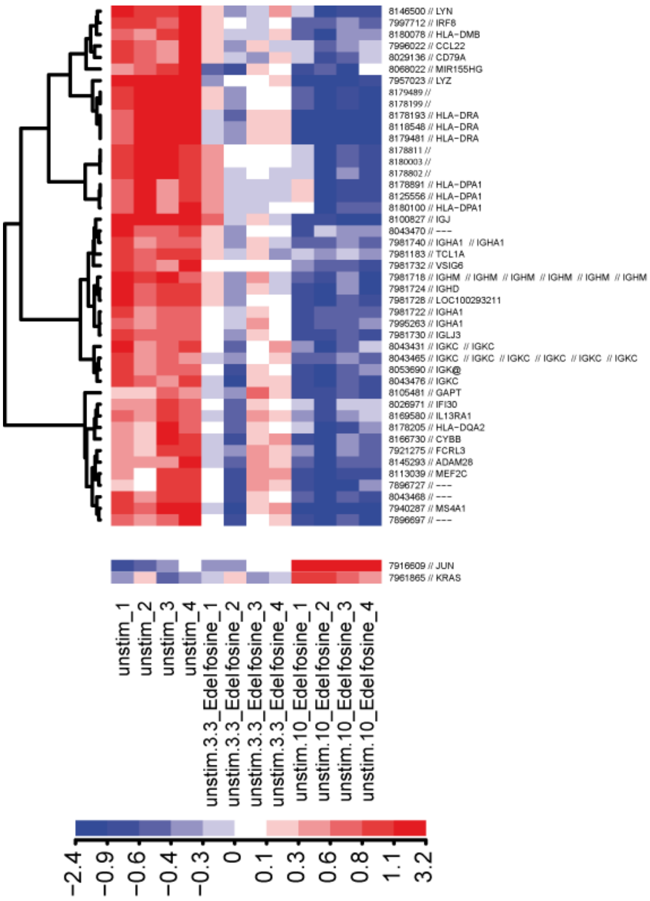

B Edelfosine-mediated increased expression of type I interferon-associated genes

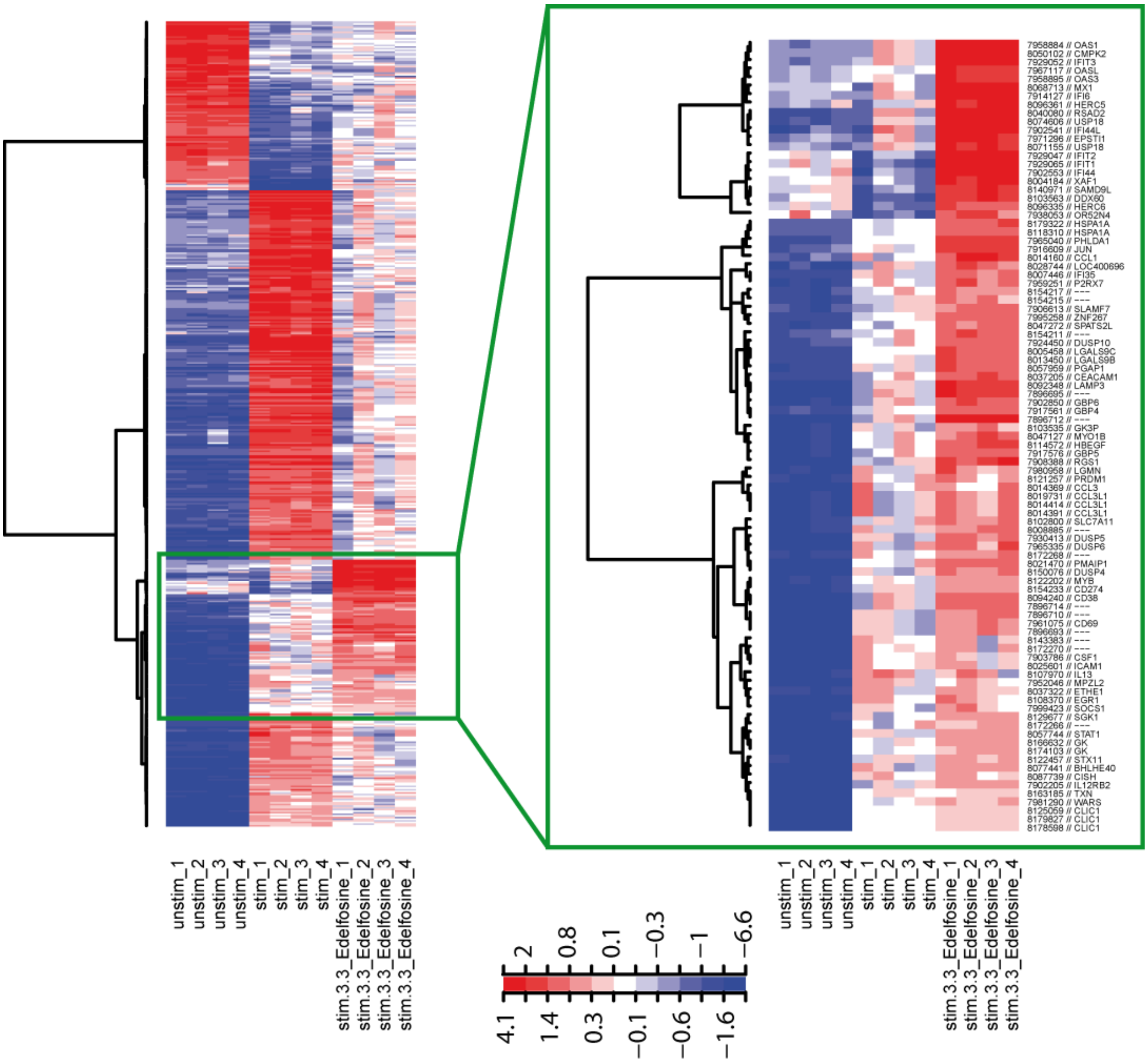

Supplement: Figure S3 — Modulation of gene expression in human CD4+ T cells mediated by stimulation and edelfosine addition. (A) The incubation of cells in absence of a stimulus resulted in an edelfosine concentration-dependent downregulation of antigen processing- and presentation-associated genes. (B) The activation of cells in presence of 3.3 µg/ml edelfosine resulted in a consistent upregulation of immune and virus response-associated genes. The values of differential gene expression changes correspond to the SLR (red for upregulation, blue for downregulation, depicted as median-centered log2-signals). Genes are clustered hierarchically in the dendrogram over the expression matrix. The height of the branches is inversely proportional to the degree of neighborhood between clusters (images generated with R statistical platform 2.12, gplots package 2.8.0). Sample size n = 4 (two male and two female age-matched donors), adjusted P-value for significant genes after t-test analysis: P<0.01. (PDF) [file pone.0091970.s003.pdf]
