# Supplementary material for: The Orally Available, Synthetic Ether Lipid Edelfosine Inhibits T Cell Proliferation and Induces a Type I Interferon Response
Source: PLoS One. 2014 Mar 25;9(3):e91970. doi: 10.1371/journal.pone.0091970 (PMC3965404; doi:10.1371/journal.pone.0091970)
Supplement: Table S1 — Summary of genes related to MHC class II, antigen processing and presentation, and immunoglobulin/B cell function (A) and genes related to immune response and response to virus (B). (DOCX) [file pone.0091970.s004.docx]

**Supplementary Table S1**

Summary of genes related to MHC class II, antigen processing and presentation, and immunoglobulin/B cell function (A) and genes related to immune response and response to virus (B).

A unstimulated + 10 µg/ml edelfosine vs. unstimulated: HLA class II-associated genes

| **Gene ID** | **Description** | **Signal Log Ratio** |
| --- | --- | --- |
| HLA-DRA | major histocompatibility complex, class II, DR alpha | -2.7 |
| IGJ | immunoglobulin J polypeptide, linker protein for immunoglobulin alpha and mu polypeptides | -2.7 |
| LYN | tyrosine-protein kinase Lyn | -1.8 |
| IGHM | immunoglobulin heavy constant mu | -1.7 |
| IGHD | immunoglobulin heavy constant delta | -1.7 |
| MEF2C | myocyte enhancer factor 2C | -1.6 |
| IGKC | immunoglobulin kappa constant | -1.6 |
| HLA-DMB | major histocompatibility complex, class II, DM beta | -1.5 |
| IGLJ3 | immunoglobulin lambda joining 3 | -1.5 |
| IRF8 | interferon regulatory factor 8 | -1.4 |
| HLA-DPA1 | major histocompatibility complex, class II, DP alpha 1 | -1.4 |
| IGK@ | immunoglobulin kappa locus | -1.4 |
| VSIG6 | V-set and immunoglobulin domain containing 6 | -1.4 |
| IGKC | immunoglobulin kappa constant | -1.3 |
| HLA-DQA2 | major histocompatibility complex, class II, DQ alpha 2 | -1.2 |
| IGHA1 | immunoglobulin heavy constant alpha 1 | -1.2 |
| FCRL3 | Fc receptor-like 3 | -1.2 |
| IL13RA1 | interleukin 13 receptor, alpha 1 | -1.1 |
| CD79A | B-cell antigen receptor complex-associated protein alpha chain | -1.1 |
| IFI30 | interferon, gamma-inducible protein 30 | -1.0 |
| CD74 | HLA class II histocompatibility antigen gamma chain | -1.0 |
| BLNK | B-cell linker | -0.9 |
| HLA-DMA | major histocompatibility complex, class II, DM alpha | -0.9 |
| IGHV4-31 | immunoglobulin heavy variable 4-31 | -0.9 |
| BTK | Bruton agammaglobulinemia tyrosine kinase | -0.8 |

B stimulated + 3.3 µg/ml edelfosine vs. stimulated: type I interferon-regulated genes

| **Gene ID** | **Description** | **Signal Log Ratio** |
| --- | --- | --- |
| IFIT2 | interferon-induced protein with tetratricopeptide repeats 2 | 4.1 |
| IFI44 | interferon-induced protein 44 | 3.4 |
| RSAD2 | radical S-adenosyl methionine domain containing 2 | 3.4 |
| IFI44L | interferon-induced protein 44-like | 3.3 |
| IFIT1 | interferon-induced protein with tetratricopeptide repeats 1 | 3.3 |
| IFIT3 | interferon-induced protein with tetratricopeptide repeats 3 | 3.3 |
| DDX60 | DEAD (Asp-Glu-Ala-Asp) box polypeptide 60 | 2.8 |
| DDX60L | DEAD (Asp-Glu-Ala-Asp) box polypeptide 60-like | 2.8 |
| MX1 | interferon-induced GTP-binding protein Mx1 | 2.6 |
| OAS1 | 2',5'-oligoadenylate synthetase 1, 40/46kDa | 2.6 |
| XAF1 | XIAP associated factor 1 | 2.5 |
| IFI6 | interferon, alpha-inducible protein 6 | 2.3 |
| OAS3 | 2'-5'-oligoadenylate synthetase 3, 100kDa | 2.0 |
| MX2 | interferon-induced GTP-binding protein Mx2 | 1.9 |
| OASL | 2'-5'-oligoadenylate synthetase-like | 1.8 |
| RGS1 | regulator of G-protein signaling 1 | 1.8 |
| IFIH1 | interferon induced with helicase C domain 1 | 1.6 |
| TNFSF13B | tumor necrosis factor (ligand) superfamily, member 13b | 1.3 |
| ISG20 | interferon stimulated exonuclease gene 20kDa | 1.4 |
| IRF7 | interferon regulatory factor 7 | 1.0 |
| EIF2AK2 | interferon-induced, double-stranded RNA-activated protein kinase | 0.9 |
| TNFSF10 | TNF-related apoptosis inducing ligand TRAIL | 0.8 |
| IL-8 | interleukin 8 | 0.6 |
| ISG15 | interferon-stimulated protein, 15 kDa | 0.6 |

(A) Unstimulated human CD4^+^ T cells were cultured with 10 µg/ml edelfosine. 25 downregulated genes were assigned to MHC class II, antigen processing and presentation, and immunoglobulin/B cell function (SLR ≤ -0.8). (B) Stimulated, 3.3 µg/ml edelfosine-treated CD4^+^ T cells demonstrated upregulation of type I IFN-associated genes involved in biological processes of immune response and response to virus (SLR ≥ 0.6).
