# Supplementary material for: The Orally Available, Synthetic Ether Lipid Edelfosine Inhibits T Cell Proliferation and Induces a Type I Interferon Response
Source: PLoS One. 2014 Mar 25;9(3):e91970. doi: 10.1371/journal.pone.0091970 (PMC3965404; doi:10.1371/journal.pone.0091970)
Supplement: Table S2 — Summary of means and corresponding SEM values for B cells (A) and T cells (B). Cell types were analyzed to determine the effect of edelfosine treatment on HLA-DR/DP/DQ expression. (DOCX) [file pone.0091970.s005.docx]

**Supplementary Table S2**

Summary of means and corresponding SEM values for B cells (A) and T cells (B).
Cell types were analyzed to determine the effect of edelfosine treatment on
HLA-DR/DP/DQ expression.

**A** Means and SEM values of B cell subtypes

| **B cells** |  | **CD19^+^** | **IgD^+^CD27^-^** | **IgD^+^CD27^+^** | **IgD^-^CD27^+^** |
| --- | --- | --- | --- | --- | --- |
|  |  |  |  |  |  |
| no edelfosine | mean: | 35925.3333 | 39288.6667 | 48650.6667 | 11177.6667 |
|  | SEM: | 7661.41306 | 7536.36058 | 6506.54348 | 2766.47539 |
|  |  |  |  |  |  |
| 3.3 µg/ml edelfosine | mean: | 31137.3333 | 34269.3333 | 40622.3333 | 10128.3333 |
|  | SEM: | 7514.20491 | 7382.89768 | 7282.40831 | 540.549206 |
|  |  |  |  |  |  |
| 10 µg/ml edelfosine | mean: | 21210.6667 | 22648.6667 | 28918.6667 | 6623.33333 |
|  | SEM: | 5378.95883 | 5379.03697 | 5689.14412 | 2832.65555 |

**B** Means and SEM values of CD4^+^ and CD8^+^ T cell subtypes

| **CD4^+^ T cells** |  | **CD27^+^CD45RA^+^** | **CD27^+^CD45RA^-^** |
| --- | --- | --- | --- |
|  |  |  |  |
| no edelfosine | mean: | 17 | 46.6666667 |
|  | SEM: | 2.51661148 | 6.64161962 |
|  |  |  |  |
| 3.3 µg/ml edelfosine | mean: | 12.6666667 | 29 |
|  | SEM: | 6.74124947 | 13.6503968 |
|  |  |  |  |
| 10 µg/ml edelfosine | mean: | 5.33333333 | 15.3333333 |
|  | SEM: | 2.72845092 | 7.68837506 |
|  |  |  |  |
| **CD8^+^ T cells** |  | **CD27^+^CD45RA^+^** | **CD27^+^CD45RA^-^** |
|  |  |  |  |
| no edelfosine | mean: | 48 | 142 |
|  | SEM: | 20.4287379 | 33.2916406 |
|  |  |  |  |
| 3.3 µg/ml edelfosine | mean: | 29.6666667 | 107.333333 |
|  | SEM: | 21.1765069 | 28.8463362 |
|  |  |  |  |
| 10 µg/ml edelfosine | mean: | 15 | 57.3333333 |
|  | SEM: | 8.66025404 | 16.3842743 |
